# Large-scale online semantic indexing of biomedical articles via an ensemble of multi-label classification models

**DOI:** 10.1186/s13326-017-0150-0

**Published:** 2017-09-22

**Authors:** Yannis Papanikolaou, Grigorios Tsoumakas, Manos Laliotis, Nikos Markantonatos, Ioannis Vlahavas

**Affiliations:** 1 Department of Computer Science, Aristotle University, Thessaloniki, 54124 Greece; 2 Atypon, 5201 Great America Parkway Suite 510, Santa Clara, 95054 CA USA; 3 Atypon Hellas, Dimitrakopoulou 7, Athens, 15341 Greece

**Keywords:** Semantic indexing, Multi-label ensemble, Machine learning, BioASQ, Supervised learning, Multi-label learning

## Abstract

**Background:**

In this paper we present the approach that we employed to deal with large scale multi-label semantic indexing of biomedical papers. This work was mainly implemented within the context of the BioASQ challenge (2013–2017), a challenge concerned with biomedical semantic indexing and question answering.

**Methods:**

Our main contribution is a MUlti-Label Ensemble method (MULE) that incorporates a McNemar statistical significance test in order to validate the combination of the constituent machine learning algorithms. Some secondary contributions include a study on the temporal aspects of the BioASQ corpus (observations apply also to the BioASQ’s super-set, the PubMed articles collection) and the proper parametrization of the algorithms used to deal with this challenging classification task.

**Results:**

The ensemble method that we developed is compared to other approaches in experimental scenarios with subsets of the BioASQ corpus giving positive results. In our participation in the BioASQ challenge we obtained the first place in 2013 and the second place in the four following years, steadily outperforming MTI, the indexing system of the National Library of Medicine (NLM).

**Conclusions:**

The results of our experimental comparisons, suggest that employing a statistical significance test to validate the ensemble method’s choices, is the optimal approach for ensembling multi-label classifiers, especially in contexts with many rare labels.

## Background

### Introduction

MEDLINE is the premier bibliographic database of the National Library of Medicine (NLM) of the United States. In June 2017 MEDLINE contained over 27 million references to articles in life sciences with a focus on biomedicine. Each of these articles is manually indexed by human experts with concepts of the MeSH (Medical Subject Headings) ontology (also curated by NLM), such as *Neoplasms*, *Female* and *Newborn*. This manual indexing process entails significant costs in time and money. Human annotators need on average 90 days to complete 75% of the citation assignment for new articles [1]. For a publication with novel and important scientific results, the first period of its lifetime is quite important, yet it is in this period that the publication remains semantically invisible. For instance, if a researcher is searching for a particular MeSH term (e.g. *Myopathy*), he/she will not be able to retrieve the latest non-indexed articles that are related to this term, if they do not contain it literally. Moreover, the average indexing cost for an article is $9.40^1^.

**Fig. 1:**
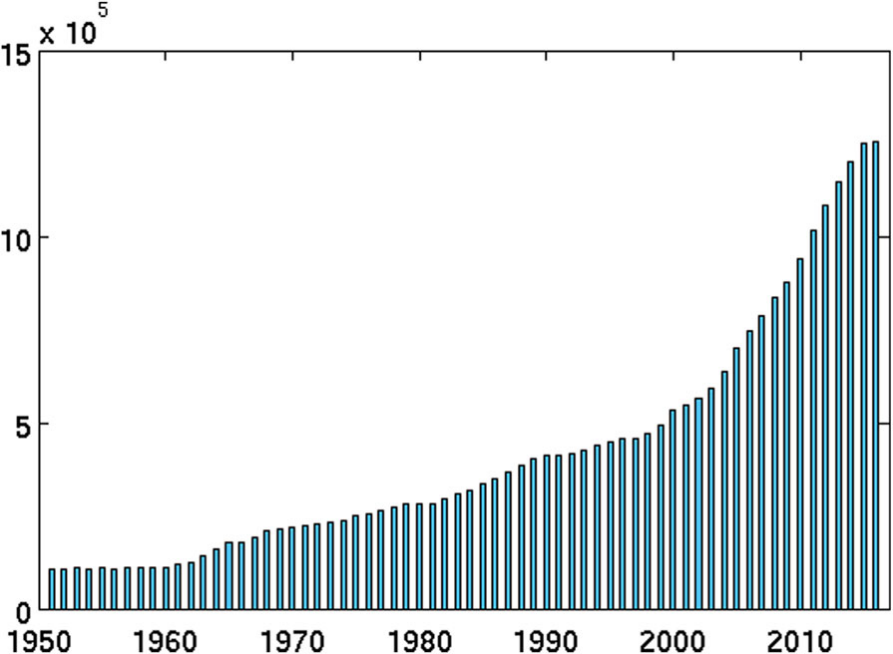
Number of articles being added to MEDLINE each year from 1950 to 2017

MEDLINE’s demand in manual indexing is steadily increasing as evident from Fig. 1, which plots the number of articles being added to MEDLINE each year from 1950 to 2017. At the same time, the available indexing budget at NLM is flat or declining. This highlights the importance of tools for automatic semantic indexing of biomedical articles. Such tools can help increase the productivity of human indexers by recommending them a ranked list of MeSH descriptors relevant to the article they are currently examining. In addition, such tools could replace junior indexers (not senior revisers) for journals where these tools achieve a high level of accuracy. Both usages of such tools are currently adopted by NLM.

From a machine learning perspective, constructing an automatic semantic indexing tool for MEDLINE poses a number of important challenges. First of all, there is a large number of training documents and associated concepts. In 2017, MeSH contained 28,489 descriptors, while PubMed contained over 27 million annotated abstracts. Efficient yet accurate learning and inference with such large ontologies and training sets is non-trivial. Additionally, MEDLINE is growing at a non-trivial rate of more than one million articles per year, i.e. more than 100 articles per hour. This calls for learning algorithms that can work in an *online* fashion both in the sense of handling additional training data as well as in the sense of being efficient enough during prediction in order to cope with the fast rate that new articles arrive. Furthermore, MEDLINE contains abstracts from about 5000 journals covering very different topics. This increases the complexity of the target function to be learned, as concepts may be associated with different patterns of word distributions in different biomedical areas.

**Fig. 2:**
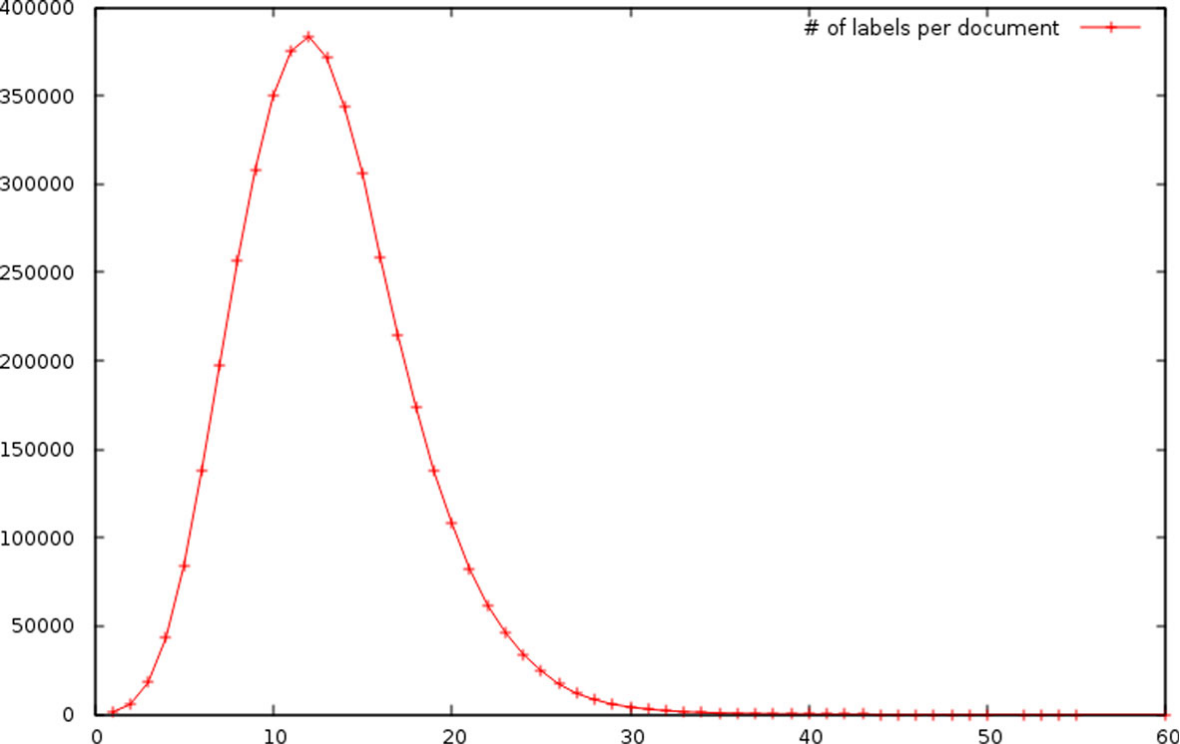
Labels per document for a subset of 4.3 million references of MEDLINE

MeSH concepts are hierarchically structured as a directed acyclic graph indicating subsumption relations among parent and child concepts. This structure is quite complex, as it comprises 16 main hierarchies with depths up to 12 levels and many children nodes belong to more than one ancestors and to more than one of the main hierarchies. While some progress has been recently achieved on exploiting such relationships, it is not entirely clear when and how these relationships help accuracy. As MeSH evolves yearly on par with the medical knowledge it describes, automatic indexing models must deal with such changes, both explicit (i.e. addition, deletion, merging of concepts) and implicit (i.e. altered semantics of concepts) ones. Also, each scientific document is typically annotated with several MeSH concepts. Such data are known as multi-label [2] and present the additional challenge of exploiting label dependencies to improve accuracy. Figure 2 shows the distribution of the number of labels per document which is Gaussian with a mean of about 13 labels per document and a heavy tail on the right.

**Fig. 3:**
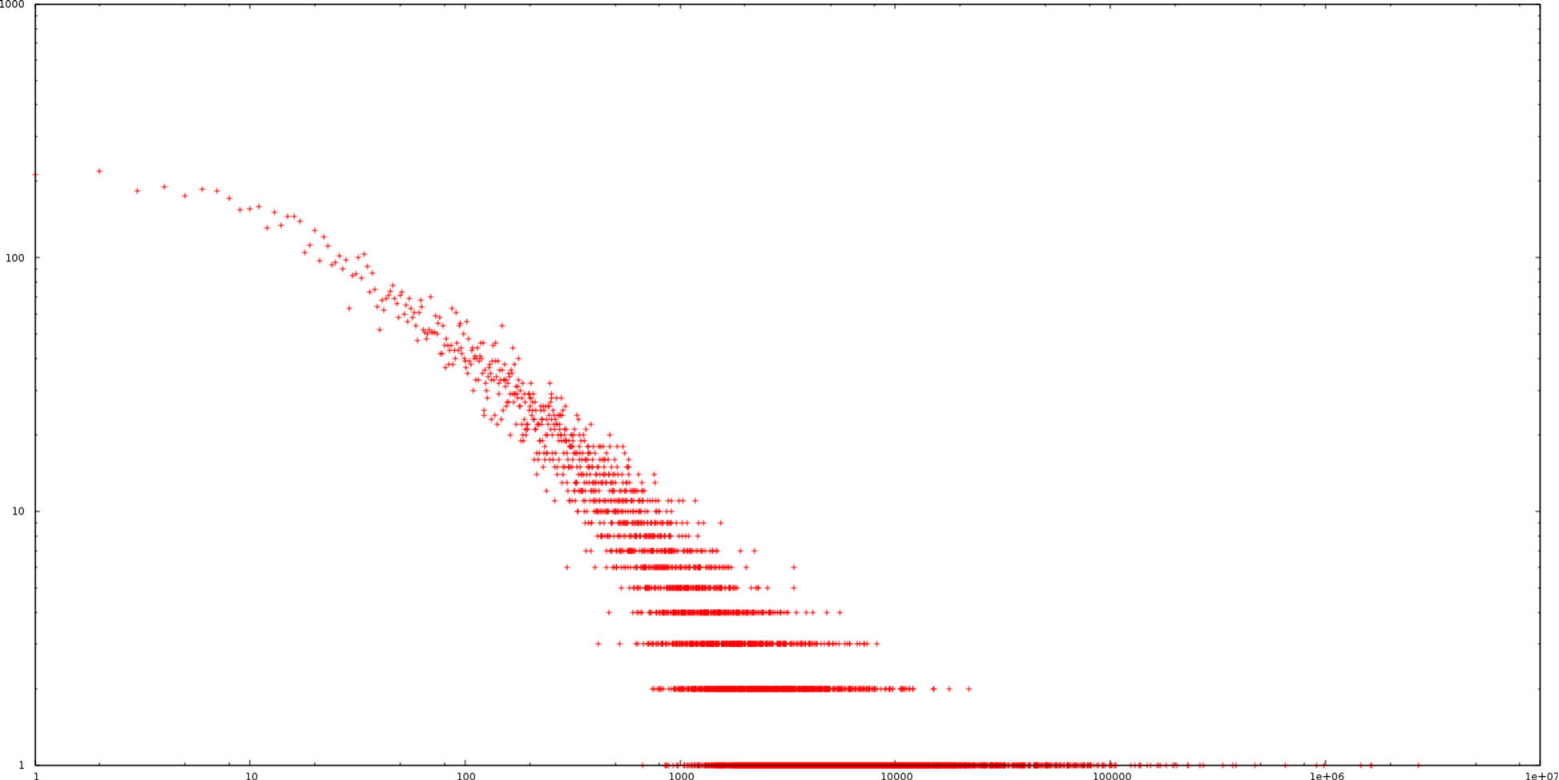
MeSH concept frequencies for a subset of 4.3 million references of MEDLINE

The distribution of positive and negative examples for most of the MeSH concepts is very imbalanced [3]. Figure 3 plots the frequencies of labels (x-axis) versus the number of labels having such frequency (y-axis) for a subset of 4.3 million references of MEDLINE. By employing the Kolgomorov-Smirnov test as proposed in [4] it can be seen that the data fits to the power law distribution with a significance level of 0.02. Less than half of the labels appearing in this subset (10,352 out of 26,509) have more than 500 positive examples and only 811 labels have more than 10,000 examples. This extreme imbalance and more precisely the fact that most MeSH labels have very few positive instances, greatly hinders learning an effective model for their automatic prediction.

The European project BioASQ [5] has organized five challenges on large-scale online biomedical semantic indexing, from 2013 to 2017, focusing on MEDLINE’s indexing problem. The official results of the five BioASQ challenges (2013–2017) are publicly available^2^. The challenge runs for fifteen weeks every year. Every week the participants are given a set of new, unannotated articles (∼ 5000) and should provide annotation for them within 21 hours. Evaluation is performed on a variety of flat and hierarchical performance measures. Our team achieved the first place in 2013 and the second place in years 2014–2017, surpassing in all cases the accuracy of the current production system of NLM, MTI.

In this paper, we present our efforts to deal with this task, from a purely machine learning perspective. The main contribution is a new multi-label ensemble method that incorporates a statistical test to combine the constituent models. As a secondary contribution, we describe the adaptation and application of already existing supervised learning algorithms to such a demanding task and a short study on the concept drift within the corpus. The rest of the paper is organized as follows: In “Multi-label ensemble methods” section we present some prior work in the field of multi-label ensemble methods and explain the differences to our method. “Methods” section describes our proposed multi-label ensemble method, MULE, and “Results” section contains the experiments, the results and a study on the concept drift that exists within the corpus. Finally, in “Discussion” section we discuss the implications of our findings and in “Conclusion” section we present the conclusions from our work with some possible future directions.

### Multi-label ensemble methods

The area of multi-label learning [2] is closely related to that of ensemble methods [6], as the most basic multi-label learning method called *binary relevance* (BR) involves learning an ensemble of binary models, one for each label. Pairwise techniques for multi-label learning, such as [7], also involve learning an ensemble of binary models. Here, however we focus on *multi-label ensemble methods*, in the sense of methods that combine multiple predictions for *all* labels, i.e. multiple rankings of labels, multiple bipartitions of the set of labels into positive and negative ones for an instance, or even multiple joint distributions for all labels.

Ensemble methods can offer improvements compared to a single model in the following cases where a single model fails to deliver a good approximation of a true hypothesis [6]: a) insufficient training data (statistical reason), b) non-convex search space with multiple locally optimal solutions (computational reason), c) the searched hypothesis space does not include the true hypothesis (representation reason). The problem that we are dealing with in this paper, fits in the first two of the above cases. First, the distribution of label frequencies follows the power law, i.e., the vast majority of the labels have very few positive instances. This means that for most labels there are no sufficient positive training instances, greatly impeding effective training of a learning model. Second, we deal with a large-scale multi-label task (∼ 10^4^ labels), which, taking into account the interactions and dependencies among labels, leads to a complex search space with multiple locally optimal solutions.

An ensemble is called *homogeneous* if all its component models emerge from the same theory, e.g., an ensemble of Support Vector Machines (SVM) classifiers. When an ensemble consists of different types of models (e.g an ensemble with SVM and Naive Bayes models), then it is called *heterogeneous*. Multi-label ensembles can be considered homogeneous if they combine models derived from the same multi-label learning algorithm *and* the same underlying single-learning algorithm in the case of problem transformation methods, e.g. an ensemble of BR models, all trained using SVMs.

There are three main approaches to combine the decisions of an ensemble’s models: (i) *selection*, where a single model is used, (ii) *fusion*, where all models are used, and (iii) *ensemble pruning*, where a subset of the models is used. In multi-label ensembles, decision combination could further be characterized as *global* if the same combination is used for all labels (e.g. the same subset of multi-label models is selected for all labels), or *local* if a different combination can be used for each label (e.g. models are fused with different weighting for each label).

In existing ensemble approaches in the literature, the authors of [8] proposed an Ensemble of Classifier Chains (ECC) in which multiple Classifier Chains (CC) are trained to model the label correlations and then they are combined through a simple global voting scheme. The Ensemble of Pruned Sets (EPS) [9] represents another approach with similar philosophy, the PS constituent models being combined again through a simple global voting scheme. In [10] three hierarchical ensemble methods are introduced in order to deal with the gene function prediction problem; Hierarchical Top-Down (HTD), Hierarchical Bayesian (HBAYES) and Hierarchical True Path Rule (HTPR) along with their cost-sensitive versions. Here different predictions are due to heterogeneous data representation and voting is again used for combining the models.

An ensemble of Bayesian Networks is proposed in [11], combining multiple joint distributions for all labels by means of their geometric average. Tahir et al. [12] present a fusion method where the probabilistic outputs of heterogeneous classifiers are averaged and the labels above a threshold are chosen. Finally, Yepes et al. [13] propose a classifier selection scheme based on the F-measure. For each label and for each of the classifiers the F-measure is computed and the best performing one is chosen to predict that particular label. Table 1 presents the aforementioned methods and classifies them. The mUlti-label ensemble method presented in this paper, described later on in “Statistical significance MUlti-Label Ensemble (MULE)” section, is also included in order to make more evident the differences among the approaches.

**Table 1:** Characteristics of the aforementioned multi-label ensemble methods and MULE

Ensemble method	Composition	Combination	Combination
		scheme	level
ECC [8]	Homogeneous	Fusion	Global
EPS [9]	Homogeneous	Fusion	Global
HTD, HBAYES,	Homogeneous	Fusion	Global
HTPR [10]			
Bayesian Networks	Homogeneous	Fusion	Global
Ensemble [11]			
Tahir et al. [12]	Heterogeneous	Fusion	Gobal
Yepes et al. [13]	Heterogeneous	Selection	Local
MULE	Heterogeneous	Selection	Local

The method proposed in this paper is closely related to [12, 13] in the sense that in all three cases, based on a validation data set, the ensemble combines directly the various models’ prediction outputs without entailing any training or interfering with the constituent models’ structure and background. This is particularly useful when wishing to combine models emerging from very different theories. Moreover, it ensures scalability of the method particularly when dealing with such large data. For instance, we tried to employ stacked BR [14] as well to our problem but without any success due to scalability issues. Regarding these three methods ([12, 13] and ours), it should be noted that [12] proposes an ensemble limited to combine only models with probabilistic outputs (i.e. outputs ranging between 0 and 1) and thus it is not appropriate for our case where models with diverse outputs need to be combined. Therefore, in the experiments presented in “Experiments” section our method is compared only to the one by [13] along with a more simplistic version of MULE.

## Methods

### Pre-processing of the data

The BioASQ corpus is a subset of a large collection of biomedical papers (∼ 12 million abstracts) curated by the NLM, through the PubMed framework. For each document, only the abstract is provided along with some other information (title, journal, year and the MeSH terms).

From the entire corpus we have considered only abstracts belonging to the journals covered by the test set, resulting to a subset of 4.3 million abstracts. Since every new article will belong to a journal and since this meta-information will always be available, it is valid to use this information for training. Moreover, this approach was motivated from the fact that different journals are expected to follow different data and label distributions and we desire the model to draw its respective training data from a data distribution as similar as possible to the data that is going to be predicted. Therefore, selecting only those abstracts that belong to the test data journal list for training, is expected to improve performance. One alternative way to think about that relates to the process of manual indexing: a human annotator will be assigned papers coming from a specific scientific field and therefore list of journals, rather than annotating papers from any field, randomly, since he will be specialized on a specific field.

The following steps in the pre-processing of the data included removal of duplicate instances, concatenation of abstract and title for every instance, removal of stop-words and selection of word tokens and pairs of word tokens (bi-grams) as features. Word tokens and bi-grams with less than five occurrences were omitted as well as those with a frequency higher than half the size of the corpus. In order to vectorize the data sets the tf-idf representation was used for the features. We furthermore performed zoning of some features, i.e., increasing the tf-idf value of features that are expected to have more influence than others in the classification task. More specifically, we multiplied the tf-idf values from *n*-grams that belonged to the title with log2 and from those being equal to some MeSH label by log1.25.

### Statistical significance MUlti-Label Ensemble (MULE)

MULE is a multi-label ensemble approach concerned with the problem of selecting the most appropriate model among its members for each different label. It assumes the existence of a heterogeneous ensemble and that different labels can be approximated better by different types of models. The standard way to approach this problem is the employment of a validation set, based on which the accuracy of each model of the ensemble is evaluated for each label.

One issue with this approach is how to compare the models based on a single label, when the goal is to optimize a global evaluation measure related to all labels whose estimation cannot be decomposed per label, such as the micro-averaged f-measure, or the example based f-measure. Comparing the models based on a local evaluation measure such as the f-measure for the particular label [13] is not guaranteed to optimize such measures. A more appropriate solution involves cyclically examining the labels, selecting the best model for each label according to the global evaluation measure until the selected models per each label do not change in two consecutive cycles. Such an approach has been followed in the past for tuning a different threshold per-label with the goal of optimizing a global evaluation measure [15].

We argue that model selection approaches based on a validation set are brittle for multi-label data streams with a large number of rare labels, like the application we are focusing on in this paper, which involves a stream of scientific articles where the label frequency distribution follows the power law. For rare labels, selection of models is untrustworthy as it is eventually made based on very little data, despite a potentially large validation set. Real-world streaming data are also often characterized by concept drift, and therefore the larger the validation set, the higher the chance that model selection will not be valid for future incoming data.

The observations above motivated the development of our approach, whose main idea is to start by trusting the globally optimal model across all labels as the best model for each label and then select a different model for a label only if it is significantly better than the global one in this label based on an appropriate statistical test. Trusting the globally optimal model is justifiable, as its evaluation is based on much more data, compared to the data for a single label. When using a statistical test to compare each other model against the globally optimal one, the choices are expected to be less optimistic and more conservative, leading to an ensemble that will be more robust to differences between the validation and test set and to the lack of enough positive samples for rare labels in the validation set.

Formally, suppose that the multi-label task to be dealt with has *L* labels, *l* being a label, and *D* documents, *d* being a document. Also, *D*_*TRAIN*_ will be the training set, while *D*_*VAL*_ and *D*_*TEST*_ the validation and test set respectively. We denote as *M* the learning models that are used and, without any loss of generality, assume that *M*_1_ is the best performing model globally, in terms of a multi-label evaluation measure. The goal is to be able to tell which of the models used is more suitable for each of those *L* labels, in terms of the same measure on some validation data set.

The general scheme for MULE then is to

1. Predict labels with all *M*_*i*_ on a validation data set
2. Determine for each label which models predict it more accurately compared to *M*_1_, i.e., which model brings an improvement with respect to a global evaluation measure.
3. Compare the differences in performance of each one of these models against *M*_1_ using a McNemar test with significance level *α* and select the one for which the null hypothesis is rejected. If the null hypothesis is rejected for more than one models, choose the one for which the null hypothesis has the lowest probability.
4. Predict accordingly on the test set for each label

In Algorithm 1 we present the pseudocode for MULE assuming the evaluation measure is the micro F-measure. Naturally, any other metric can be used instead. For instance, in the experiments we also use a variant of MULE, which optimizes the macro F-measure. In Appendix: McNemar’s statistical test we provide the implementation details for the McNemar significance test with respect to the ensemble method. The only parameter needed for MULE is the significance level *α* of the respective McNemar’s test. It should be noted that when performing multiple statistical comparisons (that is for more than two models) the family-wise error rate (FWER) should be controlled in order for the statistical comparisons to be valid. In our case, as the tests performed were parametrical, the Bonferroni-Holmes step method was used. A detailed explanation of that method is given in [16].



Initially, we also tried a similar strategy comparing classifiers in terms of their precision and recall by applying a proportion significance test (an idea based on [17]). Given two models *A* and *B* and assuming that *A* is better than *B* globally, we predict each label *l* with *A* unless if:

1. *precision*_*Bl*_>*precision*_*Al*_ and *recall*_*Bl*_>=*recall*_*Al*_ or
2. *recall*_*Bl*_>*recall*_*Al*_ and *precision*_*Bl*_>=*precision*_*Al*_,

where > means significantly better and >= means not significantly worse. If any of the above holds we predict *l* with *B*. We experimented with various confidence intervals (0.99, 0.975, 0.95, 0,90) but this approach proved to be too conservative in all cases, by allowing very few labels to be chosen from the second system, leading in some cases to negative results.

## Results

In this section we present the results obtained from our experiments. We first present the evaluation metrics used to assess performance and then we describe the data sets used in the experiments. Next, we provide the constituents models used for the ensemble methods along with their relative performance and then we present the results for the ensemble methods with the relevant discussion. In the last sub-section we present a small study on the temporal aspects of the BioASQ data.

### Evaluation measures

Through our experiments, we chose to use as a means of evaluation of performance two label-based measures that are widely used in multi-label contexts; the micro-F and the macro-F measure [2]. Our choice over other possible options (e.g. precision, recall, accuracy) is dictated by the fact that the F-measure, as well as its micro and macro variants, provide a satisfying balance between precision and recall. Moreover, the macro-F measure tends to favor rare labels whereas the micro-F tends to smooth out their effect on total performance, hence being more influenced by frequent labels. For simplicity, we provide the F, micro-F and macro-F definitions directly in terms of the true positives, false positives and false negative errors. First, let’s denote as *tp* the number of true positives of a model (i.e. the number of times an instance has a label and the model successfully assigned it), *fp* the number of false positive errors of the model (i.e. the number of times an instance does not have a label but the model assigned it erroneously) and *fn* the number of false negative errors (i.e. the number of times an instance has a label but the model did not succeed in assigning it). Equation 1 provides the F1 score used for a single-label classification problem:

1 $$ F1_{score} = \frac{2 \times tp}{2 \times tp+fp+fn}   $$

In a multi-label context such the one we deal with and given that there are *L* labels, the micro-F measure is defined as

2 $$ Micro-F_{score} = \frac{2 \times \sum_{1}^{L}{tp_{l}}}{2 \times \sum_{1}^{L}{tp_{l}}+\sum_{1}^{L}{fp_{l}}+\sum_{1}^{L}{fn_{l}}}   $$

and the macro-F respectively

3 $$ Macro-F_{score} = \frac{1}{L} \sum_{1}^{L}{\frac{2 \times tp_{l}}{2 \times tp_{l}+fp_{l}+fn_{l}}}   $$

### Data sets

We conducted experiments on two different subsets of the BioASQ corpus; data set A consists of a training set of 850,000, a validation set of 100,000 and a testing set of 50,000 documents and data set B consists of a training set of 20,000, a validation set of 20,000 and a test set of 10,000 documents. Table 2 shows the periods covered by the two data sets. The motivation behind using two different data sets in size was mainly to study how the ensemble algorithms that are tested would behave under a small training/validation set and a large one.

**Table 2:** Chronological period covered by the training, validation and test sets for both data sets

	Period
Data set A	
Training set	October 2007 - January 2012
Validation set	December 2012 - July 2013
Test set	July 2013 - January 2014
Data set B	
Training set	July 2013 - October 2013
Validation set	October 2013 - December 2013
Test set	December 2013 - January 2014

### Component models

In this section we present the algorithms that were used as components for the ensemble method, during the BioASQ challenge as well as in other experiments. Naturally, any other supervised learning model could have been used instead.

**BR SVM** We used the BR or one-vs-all approach, according which a multi-label task with *L* labels is split in *L* different binary classification problems, one for each label. A model is then trained for each one of the labels independently from the others. Although this strategy does not take into account the relations that exist among labels (e.g. hierarchies) it is particularly convenient for large-scale setups as it allows full parallelization of the training and prediction procedure. The Liblinear package [18] was used with C and e parameters at default values (1, 0.01 respectively) and a bias value of 1. The selected solver type was L2-regularized L2-loss support vector classification (L2RL2LossSVCDual). Two variations were used, one with default parameters (Vanilla) and a tuned version (Tuned). For the latter, we adjusted C = 0.33 and changed the -w1 parameter to handle class imbalance by penalizing more heavily false negative errors than false positive ones [19]. More specifically, for all labels with less than 100 positive instances in the data set the weight for the negative class is set as 1 (default value) and the weight for the positive class as

$$w_{l} = 1 + \frac{30}{pos_{l}}, \:pos_{l} = \:positive\: instances\: for\: label\: l $$

The above parameter values were chosen based on smaller scale experiments.

**Meta-Labeler** The Meta-Labeler [20] is a two-level model. It comprises a first-level multi-label learning model capable of producing a ranking of the labels per instance and a second-level model capable of predicting the number of labels per instance. We here instantiate this model as follows. We first train the Vanilla SVM models as described in the previous subsection and then predict on the new data by assigning a score to each instance-label pair, based on the distance of the instance from the hyperplane of the label’s SVM. This way, a ranking of the labels is obtained for each instance, from the most relevant one (with the highest score) to the least relevant one (lowest score). The second-order model then serves to determine automatically the number of labels per instance by employing linear Support Vector Regression (SVR). Other thresholding techniques exist in the literature, but they either require a cross-validation step which requires a long time for large data ([15]) or did not perform as well in preliminary experiments ([21]). For both levels of this algorithm the same parameters and the same feature space as for the binary models were used.

**Labeled LDA**

Labeled Latent Dirichlet Allocation (LLDA) [22, 23] is a supervised learning extension of the LDA algorithm, where each topic is equal to a label of the corpus in a one-to-one correspondence.We have used Prior LLDA [23] that incorporates prior knowledge on the labels’ distributions (i.e., their frequencies) within the training corpus. Also, we employed the CGS _*p*_ improved *ϕ* and *θ* estimators presented in [24]. We set *β*=0.01 both at training and inference and $\alpha = \frac {50}{L}$ during training and $\alpha = 50 \times \frac {f_{l}}{\sum {f_{l}}}+\frac {30}{L}$ during prediction, *L* being the number of labels and *f*_*l*_ the frequency of label *l* in the training corpus. We used one Markov chain, a burn-in of 50 iterations, a sampling lag of 5 iterations and a total of 20 samples to compute the *ϕ* parameters during training and the *θ* parameters during prediction.

### Experiments

#### Performance of the component models

In Table 3 the performance of the constituent models on data sets A and B are shown in terms of the micro-F and macro-F measures. The discriminative SVM-based models clearly outperform the probabilistic model (LLDA). More precisely, the Meta-Labeler outperforms all other models in both data sets, exhibiting a notable difference in both metrics compared to them. The prevalence of this method over the other SVM variants, particularly if we take into account the challenging properties of the BioASQ indexing task, suggests that ranking the scores of the different labels for every instance followed by some thresholding strategy is clearly more successful than the traditional classification of instances for every label (i.e. assigning 0 or 1 to an instance for every label). It should be also noted that the Meta-Labeler does not have any particular tuning to cope with the class imbalance (the base models are Vanilla SVMs), opposite to the Tuned SVMs, but still outperforms them.

**Table 3:** Performance of component models for the test sets of data sets A and B

	Micro-F	Macro-F			
Model	A	B	A	B	
Meta-Labeler	0.58555	0.49853	0.54884	0.43381	
Vanilla SVM	0.55675	0.41254	0.47891	0.35355	
Tuned SVM	0.56653	0.45631	0.51022	0.37922	
LLDA	0.36983	0.38873	0.30100	0.37140	

The second place is steadily occupied by the Tuned SVMs, which outperform their Vanilla counterparts in all cases, a finding more or less expected given the imbalanced nature of the data (we remind that the only difference between the two algorithms is that the tuned SVMs are configured to handle class imbalance, that is rare labels, by penalizing more heavily false negative errors). The Labeled LDA model is worse in all cases except for the macro-F measure in data set B, in which case it outperforms the Vanilla SVM algorithm. We should note though that we did no particular parameter tuning which seems crucial for this model. For instance, averaging over more Markov Chains for the model in data set B results in a clearly higher performance than in A which is contradictory to the fact that data set A has a much bigger training data set.

#### Comparison of the ensemble methods

As it has been stated before, the goal of an ensemble method is to achieve higher performance than its components, w.r.t. some evaluation metric. In this context, MULE in its original form seeks to optimize the micro-F measure so in the first round of experiments, MULE is compared to the method presented in [13] and to a simple version of micro-F optimization ensemble, that does not involve a statistical test (essentially this method is equivalent to MULE but omits the McNemar test). Secondly, a variant of MULE that optimizes the macro-F measure is compared to the method presented in [13]. In this case, as optimizing the F measure and the macro-F measure is equivalent, there is no third model in the comparison. In all cases, we used a significance level of 0.1 for the McNemar’s test.

During the comparisons of the ensembles, different combinations of the components were used. The motivation was to be able to capture different relations among the models and test how the ensembles would behave in this case. For instance, the Meta-Labeler is significantly better than all other models, so in this case there is an asymmetry between the components. On the other hand, the two SVM variants show rather equivalent performance. Moreover, including the LLDA model offers the possibility to test if the model can contribute in improving performance even if it is not as successful as the other components, as it comes from a different theoretical background.

Table 4 shows the micro-F measure for the algorithms on both data sets and for five different combinations of the constituent models. A △ symbol near a value indicates that the highest value (in bold) is significantly better than it at a significance level of 0.95. MULE outperforms the two other ensemble methods in all component combinations and for both data sets except for one case. The difference is statistically significant compared to the F-optimization method in all cases, but in none concerning the micro-F optimization approach. Compared to the component models, MULE is able to improve the micro-F measure in all combinations with respect to the best performing model, the difference being statistically significant for the two last combinations (*SVM*_*Tuned*_ + *SVM*_*Vanilla*_ + *LLDA* and *MetaLabeler*+ *SVM*_*Tuned*_+ *SVM*_*Vanilla*_+ *LLDA*) in data set A. In data set B, the differences are significant in all combinations except for one for MULE and the improvement is between 0.9−7.8*%*. From the two other ensemble methods, “improve-F” is better only in two cases (in data set B) compared to the component models while “improve micro-F” does so in three cases for data set A and in all cases for data set B. Odd though it may seem, “improve micro-F” and “improve F” do not seem able to benefit from the fact that the validation set is relatively large in A (100,000 instances) by demonstrating mostly negative results.

**Table 4:** Comparison of the three ensemble methods for both data sets with respect to the micro-F measure

Micro-F measure							
Data set	*MetaLabeler*	*SVM*_*Tuned*_	*SVM*_*Vanilla*_	*LLDA*	Improve micro-F	Improve F [13]	MULE
A							
	✓	✓			0.58546	0.58127 △	0.58705
	✓			✓	0.58601	0.58260 △	0.58734
			✓	✓	0.55522	0.52144 △	0.55675
		✓	✓	✓	0.57246	0.54166 △	0.57458
	✓	✓	✓	✓	0.58695	0.55836 △	0.58919
B							
	✓	✓			0.50136	0.49445 △	0.50435
	✓			✓	0.50144	0.49329 △	0.50522
			✓	✓	0.44159	0.42726 △	0.44304
		✓	✓	✓	0.46247	0.45685 △	0.45868
	✓	✓	✓	✓	0.50058	0.49227 △	0.50353

“Improve micro-F” is the initial version of MULE, without the statistical test. “Improve-F” is the method proposed by [13]. A △ symbol suggests that the difference with the best performing model is statistically significant with a z-test and a significance level of 0.05

Similarly, Table 5 shows the results for the macro-F measure on the same five combinations. In this case the MULE _Macro_ variant is used, which optimizes the macro-F measure in an identical approach to the classic MULE method. Our method outperforms the other ensemble method in all cases except for two, with the differences being statistically significant in all cases. With respect to the best performing constituent model in each combination, MULE is able to improve the macro-F measure in three combinations (*SVM*_*Tuned*_ + *SVM*_*Vanilla*_ + *LLDA*, *SVM*_*Vanilla*_+ *LLDA* and *MetaLabeler* + *LLDA*) for data set A and two combinations in data set B (*SVM*_*Tuned*_ + *SVM*_*Vanilla*_ + *LLDA*, *SVM*_*Vanilla*_+ *LLDA*), the differences being statistically significant in none of the cases. In the other cases, the ensemble is performing worse than the best performing model (Meta-Labeler). This behavior could be due to the fact that, throughout the experiments we set the *α* value for the McNemar’s test to 0.1, which is rather liberal for a statistical test. It becomes clear that there is a trade-off between the improvement that can be achieved by combining multiple models and the confidence level that we can have on this improvement. In other words, choosing a small *α* value for the statistical test is expected to lead to more reliable results but a smaller improvement over the baseline, while in the opposite case, we risk to obtain a large improvement on the validation set, that will nevertheless not be reliable (i.e. it will not be necessarily reproducible on a random test set). By completely omitting the McNemar’s test, we obtain the extreme case for the aforementioned trade-off (this is equivalent to having an *α* value of 1.0).

**Table 5:** Comparison of the three ensemble methods for both data sets with respect to the macro-F measure

Macro-F measure						
Data set	*MetaLabeler*	*SVM*_*Tuned*_	*SVM*_*Vanilla*_	*LLDA*	Improve F [13]	MULE _Macro_
A						
	✓	✓			0.53390 △	0.54820
	✓			✓	0.53221 △	0.54921
			✓	✓	0.42563 △	0.47918
		✓	✓	✓	0.49437 △	0.51099
	✓	✓	✓	✓	0.52487 △	0.54847
B						
	✓	✓			0.42573 △	0.43342
	✓			✓	0.42429 △	0.43212
			✓	✓	0.37556	0.37335
		✓	✓	✓	0.38149	0.38058
	✓	✓	✓	✓	0.42240 △	0.43324

The “improve-F” method performs worse by improving over the component baseline only in two cases, none of which is statistically significant. This is a rather interesting observation as this measure is specifically designed in order to improve the F-measure locally in every label which, as pointed out before, is equivalent to optimize the macro-F measure. These results give strong evidence for the necessity of a statistical validation of the choices an ensemble method does.

In order to study more deeply the behavior of the tested ensemble methods, Table 6 shows the number of labels that each ensemble assigns to every component model in the first series of experiments and Table 7 depicts the average frequency of labels selected by each algorithm. It is clear that the first two ensemble methods, “improve micro-F” and “improve-F”, assign a lot more labels than MULE to those models that perform worse in overall. MULE on the other hand, secures its choices on the statistical test and therefore is a lot more conservative. For instance, in the last combination of models MULE assigns only five labels to the LLDA algorithm, around two orders of magnitude less than the other two models.

**Table 6:** Comparison of the three ensemble methods regarding the number of labels predicted by each model

# of labels predicted from each model				
	*MetaLabeler*	*SVM*_*Tuned*_	*SVM*_*Vanilla*_	*LLDA*
Data set A				
Improve micro-F	10751	15002		
Improve F [13]	11256	14497		
MULE	25192	561		
Improve micro-F	19549			6204
Improve F [13]	15293			10460
MULE	25322			431
Improve micro-F			18862	6891
Improve F [13]			12900	12853
MULE			25702	51
Improve micro-F		8213	17037	503
Improve F [13]		8723	16351	679
MULE		25210	526	17
Improve micro-F	10066	2938	2499	250
Improve F [13]	10887	2815	11782	269
MULE	24814	174	760	5
Data set B				
Improve micro-F	4252	12059		
Improve F [13]	4699	11612		
MULE	16053	258		
Improve micro-F	9342			6969
Improve F [13]	10920			5391
MULE	15826			485
Improve micro-F			1500	14811
Improve F [13]			801	15510
MULE			15998	313
Improve micro-F		1804	12774	1733
Improve F [13]		1732	12688	1891
MULE		16121	38	152
Improve micro-F	3817	494	11331	669
Improve F [13]	4198	400	11053	660
MULE	15736	144	117	43

The numbers are given for the micro-F optimization (first series of experiments)

**Table 7:** Average frequency of labels for the labelsets selected by each algorithm

*Meta*−*Labeler*	16.98
*SVM*_*Vanilla*_	182.87
*SVM*_*Tuned*_	208.54
*Labeled LDA*	129.35

The results shown are for data set A and the combination of all models

In the experiments above, it could be argued that the multi-label ensemble method we propose is not improving spectacularly the component models performance. This is generally true, especially for the MULE _Macro_ variant. Nevertheless, there is some evidence that this behavior may be connected to the component models themselves and the differences in performances they have or the theoretical background they come from. For instance, the greatest improvement in terms of the micro-F metric (∼ 8*%*) is obtained for data set B when combining the two worst performing models, LLDA and Vanilla SVM, which are rather equivalent in terms of their performance and emerging from different theories. Either way, our goal in this series of experiments is to show that an ensemble method can clearly benefit from the use of a statistical test that validates it, regardless of the size of the validation data set or the nature of its component models. The fact that the two other methods, that lack this statistical validation fail largely to improve over the components, exhibiting a rather unreliable behavior (e.g. assigning many labels to worse performing models) overall, suggests strongly that a significance test is actually needed in this case. Finally, the results for the “improve-F” method indicate that optimizing locally the F-measure does not necessarily lead to an improvement over the total performance of the ensemble.

### Temporal aspects of the data

When performing a supervised learning task, the goal is usually to train a model that will fit an underlying (i.e. hidden) distribution of the data. Crucially, during this process if the new, unseen data to be predicted follows a significantly different distribution than the data used for training, the model’s performance will of course be greatly compromised. In our case, a number of factors could lead to an important change in the data distribution along time; first, the data set expands over a great period (1946–2017) and thus variations are expected in what concepts actually “mean” or, in other words, what word tokens the concepts are related to (e.g. a disease in 1990 and 2017 can be linked to totally different factors). This affects the label - word distributions and consequently the model’s performance. Another aspect are trends in science publications. Scientific papers show non-negligible trends for a particular scientific field or another. In [25] these interesting changes in trends are studied in the biological field; e.g. in 1991, 14% of the scientific papers indexed in Web of Science concerned Biochemistry while twenty years later (in 2010) this percentage has dropped to only 4%. Finally, it should be noted that NLM makes changes once every year to the MeSH ontology (i.e. the label set), a valid choice as science evolves, and the journals encapsulated by NLM change as well every year. In the 2008 MEDLINE data changes announcement for example, the NLM reports an addition of 456 new MeSH terms in the existing vocabulary^3^.

Bearing in mind the aforementioned factors we performed a short study on the concept drift, designing two experiments. For all results presented below we used the Meta-Labeler as a learning model.

**Fig. 4:**
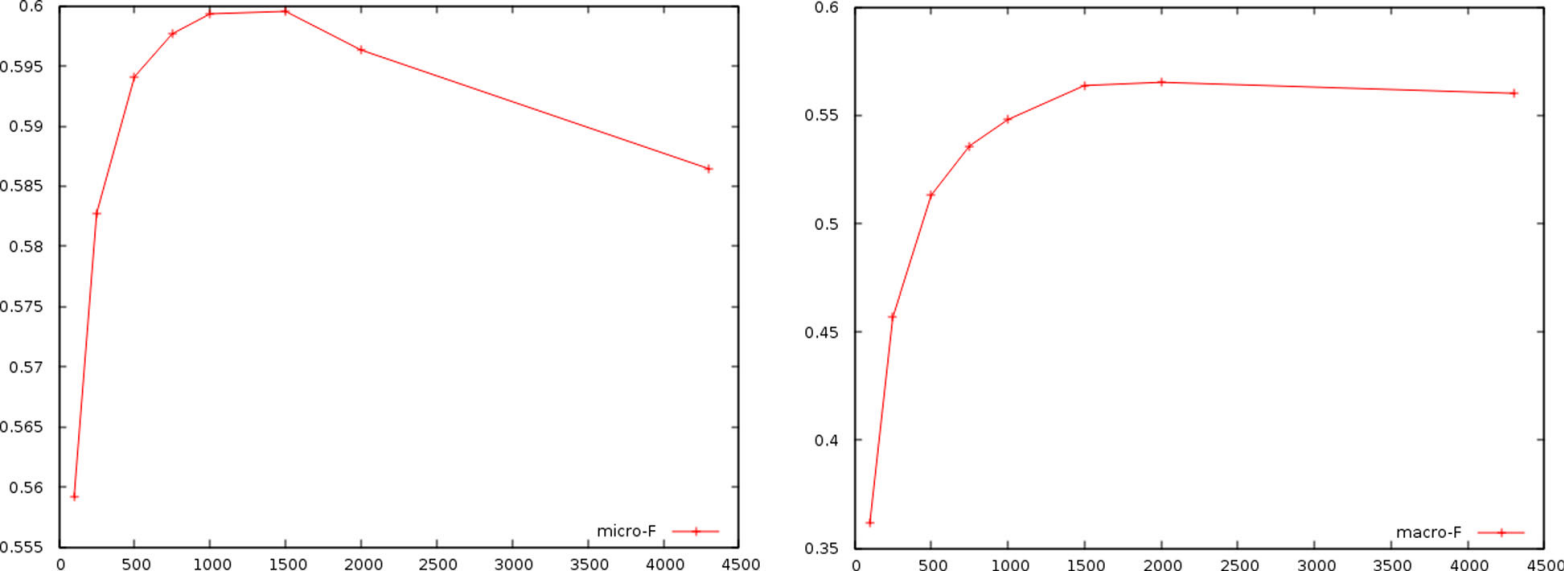
Micro-F and macro-F measures (left and right figures respectively) against number of documents (in thousands)

First, we trained classifiers with increasing training set sizes and keeping the same test set. Table 8 shows the years covered by the aforementioned training sets and Fig. 4 shows the micro and macro F-measure evolution as training sets get larger going back in time. It is easily noticeable that there is no significant gain in performance for more than 1,000,000 documents and results are even getting worse for training sets containing documents before 2004. The macro F-measure seems to have a small gain going back in time (papers from 2001), probably because it favors rare labels more than the micro F-measure and with the increase of the data set size more positive examples will be observed for them. Nevertheless, for papers before 2001 a decrease is apparent in this measure as well.

**Table 8:** Performance for training sets going back in time

Size	Date	Micro-F	Macro-F
100,000	December 2012- July 2013	0.5591	0.3616
250,000	January 2012- July 2013	0.5827	0.4567
500,000	August 2010- July 2013	0.5941	0.5130
750,000	January 2009- July 2013	0.5977	0.5358
1,000,000	August 2007- July 2013	0.5993	0.5480
1,500,000	July 2004- July 2013	0.5995	0.5637
2,000,000	August 2001- July 2013	0.5963	0.5652
4,300,000	December 1946 - July 2013	0.58646	0.56014

A fixed test set of 50k abstracts is employed for the experiment, from July 2013 to January 2014

**Fig. 5:**
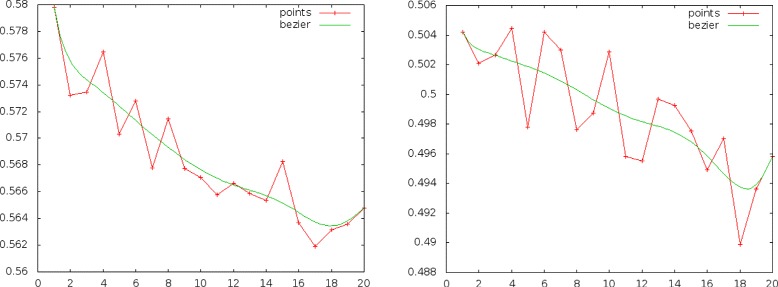
Micro-F and macro-F measures (left and right respectively) for 20 equal test sets ranging from 2007–2013

The second experiment consisted of training a classifier on 500,000 documents and then splitting the following 1,000,000 documents in 20 equal consecutive data sets to study how performance is affected as time goes by. Figure 5 shows the results. We can notice a significant drop in performance for both measures as test sets move away from the training set.

The above results, validate the presence of a non-negligible concept drift within the corpus, even for relatively small periods. Some direct conclusions are that a) it could be crucial for a learning model’s performance to choose a training data set as close (chronologically) as possible to the unseen data and b) in contrast to what usually is the case in a machine learning scenario, opting for a larger training data set can lead even to inferior results in case of the BioASQ corpus (or the PubMed corpus more generally).

## Discussion

Semantic indexing of biomedical articles represents an important and challenging task, as explained in detail in “Background” section. We have taken a novel approach by combining a number of multi-label learning algorithms in an ensemble method that validates its choices through a statistical significance test. The results that we presented, as well as the official results from five BioASQ challenges (2013–2017) in which we achieved the first place in 2013 and the second place in the following years, show that our approach can leverage the virtues of the baseline algorithms and improve over them, as well as over similar methods. This work could be extended to include additional algorithms and other variants of the multi-label ensemble, optimizing different measures or employing an alternative statistical test. Additionally, the concept drift within BioASQ and PubMed should be investigated in more depth and methods should be developed to deal with it efficiently.

## Conclusion

In this work, the different strategies that we used in order to tackle the large-scale multi-label classification problem of the BioASQ challenge were presented. Several already existing supervised learning algorithms were used, both from a discriminative and probabilistic origin. The main contribution is a multi-label ensemble that validates its choices through a statistical test. The ensemble method, MULE has been compared to two other variants in two different experimental scenarios and for different component model combinations. The results show a strong advantage of MULE over two other similar methods. Concerning the other contribution of the paper, the short study on the temporal aspects of the data, the results show a significant change of the concepts over time that should be taken into account by researchers trying to perform semantic indexing of biomedical literature. Some possible future directions of this work could include the use of additional algorithms and other variants of the multi-label ensemble. Finally, we would like to further study the concept drift within BioASQ and PubMed and experiment on methods to deal with it efficiently.

## Endnotes

^1^ http://ii.nlm.nih.gov/About/index.shtml

^2^ http://bioasq.org/participate/winners and http://participants-area.bioasq.org/results/5a/

^3^ http://www.nlm.nih.gov/pubs/techbull/nd07/nd07_medline_data_changes2008.html

## Appendix: McNemar’s statistical test

The McNemar statistical test provides a way to test differences on paired data. It is essentially a paired version of a Chi-square test. Considering the comparison of two classifiers *A* and *B*, we denote:

- *n*_00_ the number of examples correclty classified by both *A* and *B*
- *n*_01_ the number of examples correclty classified by *A* but not by *B*
- *n*_10_ the number of examples correclty classified by *B* but not by *A*
- *n*_11_ the number of examples misclassified by both *A* and *B*

The McNemar’s test is then defined as

$$\chi^{2}_{MC} = \frac{|n_{01} - n_{10}|^{2}}{n_{01} + n_{10}} $$

If *n*_01_+*n*_10_<20 the statistic is not approximated well by the chi-squared distribution. In this case the binomial distribution is used to perform an exact test. Fagerland et al. [26] have demonstrated in a series of experiments that the mid-P McNemar test is performing better than its exact-P counterpart, therefore we chose mid-P for the case of *n*_01_+*n*_10_<20:

mid-P $= 2 \sum \limits _{i=0}^{n_{01}}\binom {n_{01} + n_{10}}{i}0.5^{i}\times 0.5^{n-i}-0.5\binom {n_{01} + n_{10}}{n_{01}}0.5^{n_{01}}\times 0.5^{n_{10}}$

## Abbreviations

BR: Binary relevance
FWER: Family-wise error rate
LLDA: Labeled latent Dirichlet allocation
LDA: Latent Dirichlet allocation
MULE: Multi-label ensemble
MeSH: Medical subject heading
NLM: National library of medicine
SVM: Support vector machines
